# Relationship between Dental Occlusion and Maximum Tongue Pressure in Preschool Children Aged 4–6 Years

**DOI:** 10.3390/children9020141

**Published:** 2022-01-22

**Authors:** Yumi Sasaki, Masatoshi Otsugu, Hidekazu Sasaki, Naho Fujikawa, Rena Okawa, Takafumi Kato, Kazuhiko Nakano

**Affiliations:** 1 Department of Pediatric Dentistry, Osaka University Graduate School of Dentistry, Osaka 565-0871, Japan; yumi.15@kph.biglobe.ne.jp (Y.S.); hanahaku.sasaki@gmail.com (H.S.); rokawa@dent.osaka-u.ac.jp (R.O.); nakano@dent.osaka-u.ac.jp (K.N.); 2 Hanahaku Sasaki Dental Clinic, Osaka 538-0052, Japan; naisuigukou@gmail.com; 3 Department of Oral Physiology, Osaka University Graduate School of Dentistry, Osaka 565-0871, Japan; takafumi@dent.osaka-u.ac.jp

**Keywords:** preschool children, dental occlusion, malocclusion, muscle strength, tongue

## Abstract

Tongue function is regarded as a primary factor in the etiology of malocclusion, but details of the relationship remain unknown. The purpose of the present study was to investigate maximum tongue pressure, in preschool children to examine its relationship with dental occlusion. A total of 477 healthy children (248 boys, 229 girls, aged 4–6 years) were recruited. Dental occlusion was assessed visually to record sagittal, vertical, and transverse malocclusion, and space discrepancies. Maximum tongue pressure was measured using a balloon-based tongue pressure measurement device. Additionally, 72 children (37 boys, 35 girls, aged 4–5 years) were recruited for a 1-year follow-up study. Approximately half of the children (53.5%) showed some type of malocclusion in the present study. Maximum tongue pressure was highest in the 6-year-old children. The results of a two-way ANCOVA show that the effect of age was significant (*p* < 0.001); however, the effects of sex and dental occlusion, or the interactions among these variables, did not reach significance. Additionally, maximum tongue pressure increased significantly in the 1-year follow-up study (*p* < 0.001), especially in the normal occlusion group. Maximum tongue pressure increases markedly with growth in the preschool years and can be associated with some types of malocclusion in preschool children.

## 1. Introduction

Malocclusion is characterized by the presence of misaligned teeth and/or maxillary and mandibular discrepancies [1,2], and results from a combination of genetic influences and environmental causes during development [3,4,5]. Malocclusion in the primary dentition is thought to be one indicator for malocclusion in the permanent dentition [6,7]. The prevalence of malocclusion in the primary dentition of preschool children is reported to range from 45.5% to 83.9% based on the criteria for each study, and excessive overjet (10.2–46.1%) and deep overbite (6.05–41.5%) occur with relatively high frequencies [2,8,9,10,11,12,13,14,15,16,17].

Deleterious oral habits, such as thumb or dummy sucking, incompetent lip closure, and tongue thrusting are thought to be environmental causes leading to malocclusion [4,5,18,19,20,21]. Among them, tongue function is regarded as a primary factor in the etiology of malocclusion [4,20,22,23,24,25]. Tongue function plays an important role in various oral functions, such as mastication, swallowing, breathing, and pronunciation [26,27,28,29], and tongue dysfunction may lead to feeding difficulties, atypical swallowing, obstructive sleep apnea, and speech disorders. Therefore, it is clinically essential to objectively evaluate tongue movement in relation to malocclusion.

Tongue pressure is the force that the tongue makes against the palate. It is thought to be an objective quantitative indicator evaluated as a part of tongue movement [30,31,32,33]. In a previous study, standard values for maximum tongue pressure among adults were determined using a balloon-based tongue pressure measurement device [34]. However, evaluation of tongue pressure in children is reported to be difficult due to their limited attention span, difficulty following directions, and uncertain cooperation [35]. Nevertheless, some researchers reported that maximum tongue pressure measurements could be performed appropriately in preschool children and was related to oral function [29,36]. Additionally, the relationship between maximum tongue pressure and oral function in school-aged children has been described in some studies [31,32,33]. Recently, maximum tongue pressure was shown to be significantly lower in a skeletal class II group than in class I and class III groups in school-aged children [37]. Therefore, it is expected that tongue pressure is also associated with malocclusion in preschool children, but details of the relationship remain unknown.

The purpose of the present study was to investigate the relationship between dental occlusion and maximum tongue pressure in preschool children aged 4–6 years.

## 2. Materials and Methods

### 2.1. Ethics Statement

The present study protocol was approved by the Ethics Committee of Osaka University Graduate School of Dentistry (Approval Number H29-E39). Written informed consent was obtained from the parents of all the participants, and verbal agreement for participation was also obtained from the children.

### 2.2. Participants

A total of 583 Japanese children from one kindergarten (*n* = 470) in Osaka City and two kindergartens (*n* = 56, *n* = 57) in Takatsuki City, a suburb of Osaka City, Japan, were invited to participate in the study. The staff distributed letters outlining the purpose of the study and consent forms to the guardians of all the children. The inclusion criteria were as follows: age 4–6 years; normal language comprehension; no uncooperative behavior; no reported systemic disease; no soft tissue abnormalities. The exclusion criteria included lack of informed consent or willingness to participate in the study. A total of 477 healthy children (248 boys and 229 girls, aged 4–6 years; mean age: 5.4 ± 0.7 years) were recruited for the study. Additionally, one of the kindergartens agreed to participate in a 1-year follow-up study, and 72 children (37 boys and 35 girls, aged 4–5 years; mean age: 4.9 ± 0.3 years) agreed to participate.

### 2.3. Dental Occlusion Assessment

Dental occlusal assessment was performed as follows, based on the method proposed by the Japanese Society of Pediatric Dentistry (2015 Tokyo, Japan), with an additional assessment. In the assessment of sagittal malocclusion, anterior crossbite was defined as a negative overjet of at least 1 incisor (<3 incisors: mild; ≥3 incisors: moderate), and excessive overjet was defined as an excessive increased overjet of the most protruded maxillary incisor (≥4 mm). In the assessment of vertical malocclusion, deep overbite was defined as an excessive increased overbite (≥4 mm), and anterior open bite was defined as a lack of vertical contact between the upper and lower teeth in the anterior region. In the assessment of transverse malocclusion, posterior crossbite was defined as having at least one upper primary molar tooth occluded lingual to the buccal cusps of the corresponding lower tooth, and scissors bite was defined as having at least one upper primary molar tooth occluded buccal to the buccal surface of the corresponding lower tooth [38]. In the assessment of space discrepancies, crowding was defined as having upper primary incisor teeth or lower primary incisor teeth overlapping even slightly. Children who exhibited at least one of these conditions were classified as having malocclusion. The dental occlusal assessments were performed with the aid of a penlight, mouth mirror, and metal millimeter ruler by a pediatric dentist with 20 years of specialized experience.

### 2.4. Maximum Tongue Pressure

**Figure 1:**
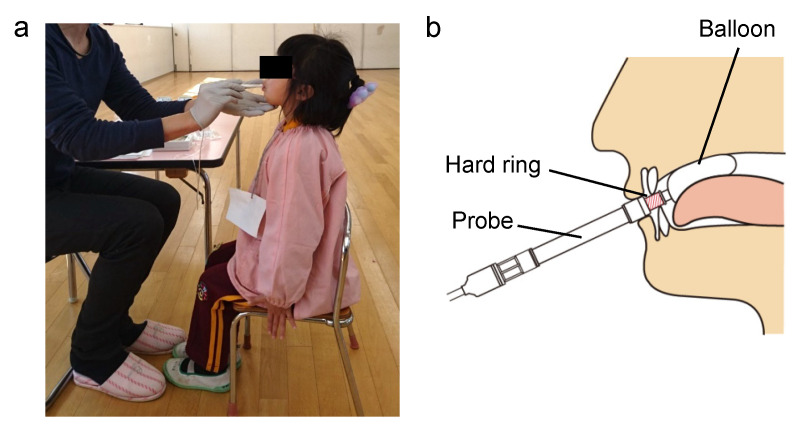
Methods of tongue pressure measurement. (**a**) Children’s posture when measuring tongue pressure. (**b**) Intraoral position of the balloon during tongue pressure measurement.

Maximum tongue pressure was measured using a tongue pressure measurement device (JMS, Hiroshima, Japan) in accordance with the method proposed in previous studies, with some modifications [32]. Initially, the instructions about the method for measuring tongue pressure were explained with a stuffed animal to make it easier for the kindergarten children to understand. The children were then placed in an upright seated position, with the Frankfort plane maintained horizontally and the soles of their feet placed flat on the floor (Figure 1a). The chairs were replaced with smaller chairs when the children’s feet did not reach the floor. The children were asked to open their mouths and the examiner put the balloon onto the anterior part of their palate. They were then asked to bite the hard ring of the probe with their upper and lower incisors. The examiner needed to ensure that the balloon was positioned vertically between the palate and the tongue (Figure 1b). The children were asked to raise their tongues and compress the balloon onto the palate for approximately seven seconds. Tongue pressure was measured (in kilopascals) using a digital voltmeter attached to a tongue pressure manometer. After practicing this procedure to improve accuracy, the measurements were recorded in duplicate, separated by an interval for rest, and the larger of the two measurements was used for analysis [20]. During measurement, children tend to lift their feet or their heels off the floor, or to hold the edge of the chair, causing their body to bend and their head to tilt forwards or backwards. Thus, when a child changed their posture during measurement, the measurement was retaken. The instructions with a stuffed animal, the chair replacements, checking of the balloon position, and the remeasurements are the revisions made in the present study that differed from the method proposed in previous studies. The measurements were recorded by a pediatric dentist with 5 years of specialized experience (who did not perform the dental occlusal examinations). Measurements were repeated after a 1-year interval for 72 of these children.

### 2.5. Statistical Analysis

Sample size was calculated using the G* Power program, version 3.1.9.6 (Franz Faul, Universitat Kiel, Germany) [39], with an anticipated correlation of more than 0.2 and a level of significance of 5%; the minimum statistical power was set to 0.8 [40]. The prevalence of malocclusion was reported by age, sex, area of residence, and the total number of children studied. The chi-square test was applied to determine the statistical associations between the independent variables and the malocclusion variables. The values of maximum tongue pressure were presented as mean ± standard deviation (SD). Tukey’s honest significance (HSD) test was performed for each age group, and Student’s *t*-tests were performed to compare the values of maximum tongue pressure by sex and dental occlusion, in each age group. Additionally, the values of maximum tongue pressure were presented by each type of occlusion in the 4–6-year age group. A two-way ANCOVA was performed to adjust for these variables. In the 1-year follow-up study of 72 children, Student’s *t*-tests were performed to analyze the increase in maximum tongue pressure. All data were analyzed using R version 3.3.3^®^ (R Foundation for Statistical Computing Vienna, Austria. URL https://www.R-project.org/, accessed on 17 January 2022), and statistical significance was set at the *p* < 0.05 level. 

## 3. Results

### 3.1. Prevalence of Malocclusion

More than half (255 out of 477, 53.5%) of the children in the present study had some type of malocclusion. A total of 62 children exhibited multiple types of malocclusion. Table 1 summarizes the prevalence of each type of malocclusion in the study population. The most frequent types were excessive overjet and deep overbite, each with a prevalence of 19.9%, followed by crowding (10.9%), anterior open bite (7.8%), and anterior crossbite (7.7%). No children exhibited transverse malocclusion, such as posterior cross bite or scissors bite, in the present study. No significant differences were found in the prevalence of malocclusion by age, sex, or area of residence.

### 3.2. Maximum Tongue Pressure

**Figure 2:**
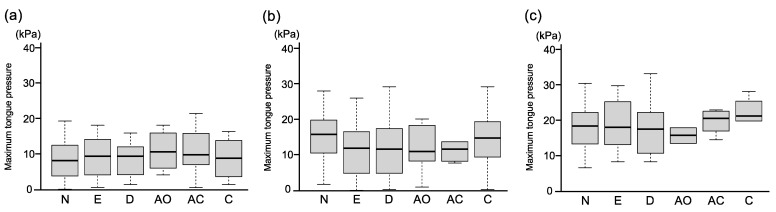
Maximum tongue pressure for each type of occlusion. (**a**): 4-year-old children, (**b**) 5-year-old children, (**c**) 6-year-old children. N; normal occlusion, E; excessive overjet, D; deep overbite, AO; anterior open bite, AC; anterior crossbite, C; crowding.

Owing to the gagging reflex, we were unable to measure maximum tongue pressure in nine children. Table 2 shows the mean values of maximum tongue pressure in 468 children (mean age: 4.9 ± 0.7 years). The highest values of maximum tongue pressure were found in the 6-year-old group (18.37 ± 6.67 kPa), followed by those of the 5-year-old group (14.18 ± 7.44 kPa) and the 4-year-old group (9.39 ± 5.42 kPa) (*p* < 0.001, in the first row in Table 2). Maximum tongue pressure of 5-year-old children with malocclusion was shown to be significantly lower than that of 5-year-old children with normal occlusion (*p* = 0.007, in the second row in Table 2). However, no significant differences in maximum tongue pressure values were found between the normal and malocclusion groups of 4-year-old and 6-year-old children. Figure 2 shows maximum tongue pressure for each type of occlusion in the three age groups. Additional analysis on excessive overjet and deep overbite revealed that, in the 5-year-old group, children with excessive overjet or deep overbite exhibited approximately 25% lower maximum tongue pressure (excessive overjet: 11.77 ± 7.13 kPa, *p* = 0.007; deep overbite: 11.93 ± 7.36 kPa, *p* = 0.004) than children with normal occlusion (15.62 ± 7.27 kPa). Additionally, no significant differences in maximum tongue pressure values were found between boys and girls in the 4−6-year age group (third row in Table 2). The results of the two-way ANCOVA reveal that the effect of age was significant (*p* < 0.001); however, the effects of sex and dental occlusion, or the interactions among these variables, did not reach significance (Table 3).

### 3.3. 1-Year Follow-Up Study in the Same Children

Table 4 shows the data for the changes in maximum tongue pressure, after a 1-year interval, for the normal occlusion and the malocclusion groups comprised of the 72 children who participated in the follow-up measurement. Compared with the baseline (8.91 ± 5.98 kPa), maximum tongue pressure increased significantly (by 64.2%) in the 1-year follow-up study (14.63 ± 6.52 kPa) (*p* < 0.001). Maximum tongue pressure increased by 89.0% in the normal occlusion group (7.54 ± 5.05), although the increase was only 42.8% in the malocclusion group (3.99 ± 6.67); the increase was significantly higher in the normal occlusion group (*p* < 0.001).

## 4. Discussion

### 4.1. Prevalence of Malocclusion

The prevalence of malocclusion in the primary dentition is reported to be diverse throughout the world, ranging from 45.5% to 83.9% [2,8,9,10,11,12,13,14,15,16,17]. In the present study, 53.5% of preschool children showed some type of malocclusion. Although the sample size of the present study (*n* = 477) was relatively small compared with previous studies (212 to 51,100), the prevalence of malocclusion in Japanese children from three kindergartens was within the above range.

The present study also showed that excessive overjet and deep overbite were associated with the highest prevalence of malocclusion. Interestingly, the prevalence of these two types of malocclusion is highly variable in other countries (excessive overjet: 10.2–46.1%, deep overbite: 6.05–41.5%), probably due to variation in the criteria used for assessing these malocclusions [2,8,9,10,11,12,13,14,15,16,17]. In this study, the prevalence of excessive overjet and deep overbite was at a lower level (approximately 20%) compared with previous studies, because more strict criteria (i.e., overbite ≥4 mm) were used. Therefore, the prevalence of excessive overjet and deep overbite might have been higher if the criteria used in the previous studies by Zhou and by Gois et al. had been adopted in the present study (i.e., excessive overjet >3 mm, overbite >3 mm) [2,8]. However, our finding that the prevalence of excessive overjet and deep overbite was higher than that of other malocclusions is consistent with previous findings [2,8,9,10,11,12,13,14,15,16,17]. No children exhibited transverse malocclusion in the present study, although several children had a lateral edge-to-edge bite, which was not included in the malocclusion criteria. This finding supports previous findings that Asians generally have a low prevalence of posterior crossbite and scissors bite [10,38]. Additionally, 62 out of 477 children exhibited two or more malocclusions. Therefore, our study sample was characterized by heterogeneity, with large variability in the number and type of malocclusion. Therefore, considering the relatively small sample size in the present study, caution is needed when interpreting further results regarding the association between malocclusion and tongue pressure.

### 4.2. Maximum Tongue Pressure

We evaluated maximum tongue pressure in 468 children aged 4–6 years. Tongue pressure was measured by examiners, rather than by the children, using a balloon-based tongue pressure measurement device. Additionally, two factors relating to the measurement of maximum tongue pressure in children were strictly standardized in the present study.

First, body and head posture were found to influence tongue position and pressure [41,42,43]. Children find it difficult to stay still during the measurement process; thus, in this study, the examiners maintained the consistency of the children’s posture, with the soles of their feet fully on the floor during measurement and the height of their chairs adjusted as needed. Second, the examiners took care to place the balloon correctly and watched for any mouth movements preventing measurement. For example, in some children whose tongue thrusting and gagging reflexes prevented the examiner from placing the balloon correctly, the examiner taught the children the correct tongue movement and asked them to open their mouth widely. Then, the examiner placed the balloon precisely between their palate and their tongue before tongue thrusting occurred.

Infantile swallowing, associated with tongue thrusting, has been identified in approximately 50% of 5-year-old children, transitioning gradually into mature swallowing towards the end of the mixed dentition period [20,44,45,46]. Tongue pressure in older adults is defined as the maximum voluntary pressure governed by their capacity [47]; however, maximum tongue pressure in children is not always voluntary during measurement using the balloon-based device. Taking these points into consideration, it is desirable that tongue pressure is measured by examiners rather than by the children until the age of approximately 12 years, to ensure that the balloon is correctly placed.

Under this careful measurement protocol, the values for maximum tongue pressure (means ranged from 9.39 ± 5.42 to 18.37 ± 6.67 kPa) were likely lower than those reported in previous studies (from 16.67 ± 7.49 to 25.38 ± 8.15 kPa) in children aged 4–6 years [36]. However, as previously reported in preschool children [29,36], maximum tongue pressure differed among age groups, but not between boys and girls.

### 4.3. Maximum Tongue Pressure and Malocclusion

The present study showed that maximum tongue pressure of 5-year-old children with malocclusion was significantly lower than that of 5-year-olds with normal occlusion. A previous study reported that school-aged children with skeletal class II occlusion exhibited a tendency towards low maximum tongue pressure [37]. Similarly, crude analysis revealed that 5-year-olds with excessive overjet and deep overbite children exhibited approximately 20% lower maximum tongue pressure than those with normal occlusion. These findings may be associated with the morphological characteristics of excessive overjet and deep overbite, such as a compromised capacity for tongue lifting [48,49] and a low tongue position [50]. However, no difference was found between the normal and malocclusion groups in the 4- and 6-year-old groups. Additionally, when adjusted for age, sex, and malocclusion, the effects and interactions with malocclusion did not reach significance in the total study sample. These results expose the limitations of cross-sectional analysis in demonstrating the association between malocclusion and tongue pressure in children aged 4–6 years; individual differences in development during the transition from primary to mixed dentition creates larger variability in the parameters and requires greater statistical power.

### 4.4. Development of Maximum Tongue Pressure

Our results show that maximum tongue pressure was higher in the older age group; that is, it was highest in the 6-year-old group. This result is consistent with those of previous cross-sectional studies showing that maximum tongue pressure correlated significantly with age in preschool children [29,36]. However, longitudinal changes in maximum tongue pressure in each individual have never been investigated. Thus, in the present study, follow-up measurements were taken from a subsample of our study population. Our results reveal that maximum tongue pressure significantly increased by 64.2% during the 1-year follow-up period, in preschool children aged 4–6 years. Compared with school children in the previous cross-sectional studies (approximately 6.3% per year, 38.0% increase in 6 years) [31], the increase is likely larger in the preschool children in the present study. Additionally, maximum tongue pressure increased by 89.0% in the normal occlusion group, while it only increased by 42.8% in the malocclusion group (*p* < 0.001). This result suggests that malocclusion in children aged 4–5 years can be associated with a smaller increase in maximum tongue pressure over a 1-year period. More follow-up studies on maximum tongue pressure in preschool children are needed, ideally with larger sample sizes and a focus on specific types of malocclusion.

### 4.5. Limitations

The present study had some limitations. The definition of malocclusion was based on the classification constructed by the Japanese Society of Pediatric Dentistry; therefore, it would be difficult to directly compare the prevalence of our results with those from other countries, which may use different malocclusion classifications and criteria. Additionally, our study sample was derived from three kindergartens in a local area of Japan, and the sample size was small. Therefore, the data do not represent the prevalence of malocclusion in all Japanese preschool children. Furthermore, the cross-sectional analysis was limited in its ability to demonstrate the association between malocclusion and tongue pressure in preschool children, and the longitudinal analysis had a small sample size. Further prospective studies on maximum tongue pressure are needed in preschool children, focusing on specific types of malocclusion. Finally, although the methodology for the measurements of tongue pressure was strictly standardized in the present study, unknown biases cannot be discounted, and oral habits that could potentially affect tongue pressure were not included in the analysis. It has long been thought that tongue volume, posture, and mobility affect the morphology of the dental arches and the dental occlusion [51]. Maximum tongue pressure can be useful for assessing the association between oral functions and malocclusion in children. However, it should be noted that a meticulous measurement protocol is necessary for preschool children. Moreover, it should be recognized that maximum tongue pressure is just one of the objective indicators for the diverse aspects of oral function.

## 5. Conclusions

Within the limitations of this experimental study, the following conclusions can be drawn. More than half of the preschool children in the present study exhibited malocclusion, and excessive overjet (19.9%) and deep overbite (19.9%) were the most frequently observed. Maximum tongue pressure increases markedly with growth in the preschool years. Although maximum tongue pressure varies among individuals, malocclusion is an important variable. We propose that maximum tongue pressure is one of the factors associated with malocclusion.

## Figures and Tables

**Table 1:** Prevalence of each type of malocclusion in Japanese children aged 4–6 years.

Occlusal Traits	4 Years Old	5 Years Old	6 Years Old	Total
	(*n* = 160)	(*n* = 217)	(*n* = 100)	(*n* = 477)
Sagittal relationship	28.8%	23.0%	36.0%	27.6%
Anterior crossbite	9.4%	6.9%	7.0%	7.7%
(mild/moderate)	(1.9%/7.5%)	(2.8%/4.1%)	(2.0%/5.0%)	(2.3%/5.5%)
Excessive overjet	19.4%	16.1%	29.0%	19.9%
Vertical relationship	26.9%	28.1%	28.0%	27.7%
Deep overbite	18.1%	22.1%	18.0%	19.9%
Anterior open bite	8.8%	6.0%	10.0%	7.8%
Transversal relationship	0%	0%	0%	0%
Posterior crossbite	0%	0%	0%	0%
Scissors bite	0%	0%	0%	0%
Space discrepancies				
Crowding	10.0%	12.4%	9.0%	10.9%

**Table 2:** Maximum tongue pressure in children aged 4–6 years.

Age	4 Years Old		5 Years Old		6 Years Old	
	(*n* = 155)		(*n* = 213)		(*n* = 100)	
Maximum tongue pressure (kPa)	9.39 ± 5.42		14.18 ± 7.44 ^a^		18.37 ± 6.67 ^a,b^	
Dental occlusion	Normal	Malocclusion	Normal	Malocclusion	Normal	Malocclusion
	(*n* = 74)	(*n* = 81)	(*n* = 103)	(*n* = 110)	(*n* = 42)	(*n* = 58)
Maximum tongue pressure (kPa)	8.77 ± 5.65	9.96 ± 5.17	15.62 ± 7.27	12.86 ± 7.41 ^c^	17.82 ± 6.49	18.72 ± 6.89
Sex	Boys	Girls	Boys	Girls	Boys	Girls
	(*n* = 81)	(*n* = 74)	(*n* = 104)	(*n* = 109)	(*n* = 59)	(*n* = 41)
Maximum tongue pressure (kPa)	10.16 ± 5.67	8.55 ± 5.04	14.47 ± 7.65	13.90 ± 7.28	18.89 ± 7.19	17.63 ± 5.85

^a^: *p* < 0.001 versus 4-year-olds; ^b^: *p* < 0.001 versus 5-year-olds; ^c^: *p* = 0.007 versus normal occlusion in 5-year-olds.

**Table 3:** Results of the two-way ANCOVA.

	Degree of Freedom	*F* Value	*p* Value
Sex	1	3.203	0.074
Occlusion	1	2.925	0.088
Age	1	39.834	<0.001
Sex: Occlusion	1	3.354	0.068
Sex: Age	1	2.939	0.087
Occlusion: Age	1	3.649	0.057
Sex: Occlusion: Age	1	3.837	0.051
Residuals	460		

**Table 4:** 1-year follow-up measurements of children in the normal occlusion group and the malocclusion group.

	Number	Age (Y)	Boy (%)	Maximum Tongue Pressure (kPa)		Increase Amount	Increase Rate
				Before	After	(kPa)	(%)
Normal	35	4.7 ± 0.3	51.4%	8.47 ± 6.05	16.01 ± 6.41	7.54 ± 5.05	89.0
Malocclusion	37	4.7 ± 0.3	51.4%	9.33 ± 5.97	13.32 ± 6.44	3.99 ± 6.67	42.8
